# Parental Attachment Types and Stress Management in a PersonOriented Context Among Hungarian Adolescents

**DOI:** 10.17505/jpor.2026.29453

**Published:** 2026-08-24

**Authors:** Karola Diósi, András Vargha

**Affiliations:** 1 Doctoral School of Psychology, Eötvös Loránd University, Budapest; 2 Institute of Psychology, Eötvös Loránd University, Budapest; 3 Institute of Psychology, Károli Gáspár University of the Reformed Church in Hungary

**Keywords:** parental attachment, stress management, ECR-RS, CISS-21

## Abstract

Adolescence is a period marked by increased stress, in which it is especially important how they manage stress. Previous research suggests that both relational contexts and individual characteristics influence coping strategies, highlighting the need to examine stress management in adolescence within a broader developmental framework. The aim of this study is to investigate the relationship between parental attachment and stress management strategies in adolescence in a person-oriented approach. The sample consisted of 1,485 Hungarian secondary school students (793 males, 687 females; five with unidentified gender), aged 16–23 years, drawn from a nationwide school-based survey. Data were analyzed using self-report measures of parental attachment (ECR-RS) and coping styles (CISS-21). Attachment profiles with four parental attachment scales were identified through *k*-means cluster analyses, and their associations with coping strategies and gender were examined. *k*-means cluster analysis based on POMS-transformed variables identified a 6-cluster solution with acceptable homogeneity and separation (EESS% > 70; HCmeanS < 1; Silhouette coefficient > 0.50). Cluster membership was significantly associated with gender (χ*^2^* = 34.86, *df* = 5, *p* < .001, Cramér’s *V* = 0.153). Parental attachment patterns are associated with distinct coping configurations in adolescence, with attachment security supporting more adaptive integration of coping strategies under stress. These findings highlight the importance of attachment-informed perspectives for understanding individual and gender-related differences in adolescent stress management.

## Introduction

Adolescence is a critical period characterized by significant physiological, psychological, and social changes (Arnett, 1999; Steinberg, 2005; Sawyer et al., 2012). During this time, stress levels often escalate due to various factors such as academic pressure, social dynamics, and the process of identity formation (Grant et al., 2003; Seiffge-Krenke, 2011). Coping mechanisms play a pivotal role in determining their psychological wellbeing and overall development (Compas et al., 2017). Coping strategies are the behaviors, thoughts, and emotions used to manage the demands of stressful situations. These strategies can be broadly categorized into adaptive and maladaptive coping mechanisms (Lazarus & Folkman, 1984; Compas et al., 2001).

Among the various influences on coping strategies, parental attachment stands out as a fundamental factor (Kobak & Sceery, 1988; Allen et al. 2018).

Parental attachment, rooted in the early relationship between a child and their caregivers, profoundly impacts emotional regulation, social competence, and stress-management in later stages of life. According to Bowlby’s attachment theory, early interactions with caregivers form internal working models that shape how individuals perceive and respond to stress throughout their lives (Bowlby, 1988).

This article aims to explore the relationship between parental attachment and coping with stress among adolescents. By examining existing literature and empirical data, our study seeks to elucidate the ways in which the type of parental attachment is related to stress responses and coping strategies among adolescents. Understanding this connection can provide insight into promoting mental health and wellbeing in this vulnerable group, guiding parents, educators, and mental health professionals in their efforts to support adolescent development.

### Parental attachment and stress management

Bartholomew and Horowitz (1991) introduced a four-category model of adult attachment that expands upon the traditional secure and insecure dichotomy. Their model includes four attachment styles: secure, preoccupied, dismissive-avoidant, and fearful-avoidant. These styles are based on two dimensions: the model of self (positive or negative) and the model of others (positive or negative).

Secure attachment, characterized by reliable and supportive parenting, fosters a sense of security and self-efficacy in children. This sense of security enables adolescents to approach stressful situations with confidence and adaptive coping strategies (Mikulincer & Shaver, 2007).

Conversely, insecure attachment, resulting from inconsistent or neglectful parenting, can lead to heightened anxiety and the adoption of maladaptive coping mechanisms. Adolescents with insecure attachment may struggle with emotional regulation and are more likely to resort to avoidance or emotion-focused coping strategies, which can exacerbate stress and negatively impact on psychological health (Kobak & Sceery, 1988).

Fraley and Shaver (2000) provided further elaboration on the dimensions of attachment, with a focus on the continuous nature of attachment-related anxiety and avoidance. Their research underscores how these dimensions impact stress coping mechanisms.

- Attachment-Related Anxiety: Individuals high in attachment-related anxiety tend to worry about rejection and feel uncertain about the availability and support of others. This anxiety can lead to hyperactivating strategies, such as excessive seeking reassurance and support during stress, which may provide temporary relief but can also reinforce feelings of insecurity.
- Attachment-Related Avoidance: Those high in attachment-related avoidance prefer emotional self-reliance and distance themselves from others. This deactivating strategy involves suppressing emotional responses and avoiding stress-related discussions, which can prevent effective stress management and lead to long-term psychological and intersocial issues.

The interplay between these attachment dimensions and coping strategies highlights the profound impact of parental attachment on stress-management (Allen et al. 2018; Fraley & Shaver, 2000).

### Coping with stressful situations among adolescents

Endler and Parker’s multidimensional coping theory provides a comprehensive framework for understanding how individuals manage stress (1990). According to their theory, coping strategies are broadly categorized into three types: task-oriented, emotion-oriented, and avoidance-oriented coping. Each strategy serves a distinct function in managing stress (Endler & Parker, 1990):

- Task-Oriented Coping: This strategy involves efforts to solve the problem or alter the situation causing stress. Adolescents employing task-oriented coping are likely to engage in active problem-solving, seek information, and take direct actions to address the source of stress. This approach is generally considered adaptive because it focuses on changing the stressor or the individual’s relationship to it, thereby reducing the impact of stress. Task-oriented coping is associated with better psychological adjustment and lower levels of distress among adolescents.
- Emotion-Oriented Coping: Emotion-oriented coping focuses on managing the emotional response to stress rather than the stressor itself. This can include strategies such as seeking emotional support, expressing emotions, and using techniques to reduce emotional distress, such as relaxation or distraction. While emotion-oriented coping can provide temporary relief, it may not address the underlying cause of stress, potentially leading to ongoing distress if the stressor remains unresolved. Adolescents using emotion-oriented coping might experience fluctuating levels of emotional distress depending on the effectiveness of their emotional regulation strategies.
- Avoidance-Oriented Coping: Avoidance-oriented coping involves efforts to evade the stressor altogether. This can be achieved through distraction, such as engaging in other activities, or social diversion, such as seeking comfort in social interactions unrelated to the stressor. While avoidance can offer immediate relief from stress, it is often considered maladaptive in the long term because it prevents individuals from addressing and resolving the stressor. Adolescents relying heavily on avoidance-oriented coping may experience prolonged stress and potential negative outcomes, such as increased anxiety and lower academic performance.

Adaptive coping strategies generally involve problemsolving and active efforts to address the stressor or one’s emotional response to it. Among these strategies, problemfocused coping, which entails directly addressing the problem to reduce or eliminate the source of stress, is considered highly effective. Adolescents who engage in problem-focused coping tend to show better psychological adjustment and lower levels of distress (Frydenberg & Lewis, 1993).

Emotion-focused coping is also an adaptive strategy involving the management of emotional responses to stress rather than the stressor itself. This can include seeking social support, practicing relaxation techniques, and reframing the situation in a more positive light. While emotion-focused coping can provide immediate emotional relief, it is most effective when used in combination with task-oriented strategies (Compas et al., 2001).

In contrast avoidance and denial can exacerbate stress and lead to poorer mental health outcomes. Avoidance coping involves efforts to evade the stressor, such as engaging in distracting activities or withdrawing from the situation. Although avoidance can provide temporary relief, it often prevents the underlying problem from being resolved and can lead to increased anxiety and stress over time (Endler & Parker, 1990; Compas et al., 2001).

The efficacy of coping strategies among adolescents is influenced by various factors, including individual differences in personality, the nature of the stressor, and the available social support. Adolescents with high resilience and a strong sense of self-efficacy are more likely to employ adaptive coping strategies such as task- and/or emotion-oriented coping and exhibit better psychological outcomes. Social support from family, friends, and peers also plays a critical role in buffering against stress and promoting effective coping (Seiffge-Krenke, 2011).

Moreover, gender differences have been observed in coping strategies among adolescents. The study by Eschenbeck, Kohlmann, and Lohaus (2007) underscores significant gender differences in coping strategies among children and adolescents. Female adolescents are more likely to use emotion-focused coping, such as seeking social support and expressing emotions, whereas male adolescents tend to use more task-focused and avoidance strategies. This finding aligns with the same results from different countries (Frydenberg & Lewis, 1993; Cosway et al., 2000; Rafnsson et al., 2006; Matud, 2004).

Among female students loneliness is negatively correlated with task-focused coping and social distraction-focused coping. Girls who feel less lonely are more effective, more goaloriented in coping with challenging situations, and may even find resources in their social relationships. These results support the view that a positive family atmosphere and supportive social relationships are protective factors in coping effectively with stress (Frydenberg & Lewis, 1993; SeiffgeKrenke, 1993; Allen et al., 2018.).

## Materials and Methods

### Procedures

The original research was carried out within the framework of the sub-project “Health promotion and universal drug prevention” (IEUD) of the National Institute for Family and Social Policy, TÁMOP-7.2.1-11/K-2012-0004, “Professional methodological basis for complex, integrated approach to youth policy development”. The aim of the research was to obtain a representative picture of the mental health status of the 6–23-year-old generation at national level, including chemical substance use (alcohol, drug use and smoking) and related risk and protective factors, based on a sample of about 8000 people. Ethical approval was obtained from the Human Subjects Research Ethics Committee of Károli Gáspár Reformed Church University.

To conduct the survey, written permission was obtained from school directors. An information sheet outlining the study’s aims was given to school principals, and informed consent was secured from the students’ parents. Participants were assured of confidentiality and anonymity, with data used solely for research purposes.

### Participants

Data were used from a Hungarian study on substance use during adolescence (Mirnics et al., 2021). Our sample consisted of 1716 students, 905 male (age range: 16-23, mean age=17.7, *SD*=1.04) and 805 female (age range: 16-22, mean age=17.5, *SD*=0.92) students at secondary schools in Hungary, from grades 11. Students were recruited from all twenty counties in Hungary, representing the population of Hungary. Students filled out the questionnaire in a school setting. The analyses were restricted to students, who had usable scores on all scales of measured parental attachment (*N* = 1485, among them 793 males and 687 females, for five students gender was not identified). Of the total sample, 26 students (1.5%) did not begin filling out the parental attachment test because they were absent that day. Another 205 students (11.9%) began to fill out the form but did not complete it. The dropout was not related to gender (χ^*2*^ = 1.91, *df* = 1, *p* = .167). However, it was related to the type of settlement (χ^*2*^ = 19.59, *df* = 2, *p* < .001). This was due to a higher percentage of missing data (30.3%) among students living in smaller settlements compared to those living in the capital (15.7%) or other towns (12.1%).

Vargha and Grezsa (2024) and Vargha (2025) presented clusters of male adolescents based on their attachment styles using the same sample. However, the present study was not restricted to male students only, allowing for the attainment of more valid and generalizable results.

### Measures

#### Experiences in Close Relationships – Relationship Structures Questionnaire (ECR-RS)

The ECR-RS Questionnaire (Fraley et al., 2011) is a versatile tool used to assess attachment styles across different types of close relationships, including romantic partners, parents, and friends. It measures two main dimensions: anxiety, which pertains to fears of rejection and abandonment, and avoidance, which relates to discomfort with closeness and dependence. In the attachment model, these two key dimensions determine four main style types (Benoit, 2004; Fraley & Shaver, 2000). Individuals with secure attachment exhibit low levels of both anxiety and avoidance. Conversely, attachment becomes highly problematic in the insecureavoidant type, where both anxiety and avoidance are elevated. Those classified as insecure-resistant rejecting (ambivalent, avoiding, denying) score significantly above average on the avoidance dimension, whereas those identified as insecure-disorganized (obsessed, flooded) score significantly above average only on the anxiety dimension.

Administered as a self-report questionnaire, individuals rate their agreement with various statements on a 7-point Likert scale. 10 items (six measuring avoidance and four measuring anxiety) belonged to each of the four domains with the same questions. In our adolescent sample, the questionnaire included only 5-point Likert-scale test questions in the mother and father domains. For the Anxiety and Avoidance dimensions, subscales were created from the corresponding items. As recommended by Fraley et al. (2011), item 10 was excluded from both subscales due to significant cross-loading observed in exploratory factor analyses of the ten items within each domain. The variables of the analyses were the four avoidance (Avoid) and anxiety (Anx) scales of the mother (Mo) and father (Fa) domains of ECR-RS, called AvoidMo, AnxMo, AvoidFa, and AnxFa. These scales were calculated as the average of the corresponding five-point items. If one or more items were missing from an anxiety scale, the scale score was also considered missing. For the avoidance scales (which have six items), one item could be excluded when calculating the average from the usable items. All ECR-RS scales showed excellent reliability in the present study (see Table 2 below).

#### Coping in Stressful Situation (CISS-21)

The Coping Inventory for Stressful Situations (CISS-21) is a shortened form of the original 48-item CISS (Endler & Parker, 1990), designed to assess individuals’ habitual coping tendencies when facing stress. The instrument is grounded in Endler and Parker’s multidimensional conceptualization of coping, which distinguishes between cognitive–behavioral strategies aimed at problem solving, emotional regulation, and interpersonal diversion. The questionnaire contains the following three subscales:.

- task-oriented coping (TaskOri, 7 items), reflecting active problem solving and efforts to alter the stressful situation;
- emotion-oriented coping (EmotOri, 7 items), capturing self-focused emotional responses such as rumination or emotional distress;
- avoidance-oriented coping (AvoidOri, 7 items), indicating the tendency to cope by engaging in social interactions that provide distraction or treat-oneself distraction coping, reflecting the use of pleasurable or distracting activities to regulate stress.

Items are rated on a 5-point Likert scale, with higher scores representing stronger reliance on the respective coping strategy. This abbreviated, 21-item version is widely used in adolescent and adult populations due to its brevity and good psychometric properties (Calsbeek et al., 2003; Boysan, 2012; Imran et al., 2020). In the present study all CISS-21 scales showed acceptable reliability measured by Cronbach’s α (TaskOri: 0.811; EmotOri: 0.825; AvoidOri: 0.696) and McDonald’s ω (TaskOri: 0.812; EmotOri: 0.829; AvoidOri: 0.684). These scales were calculated as the average of the corresponding five-point items.

In addition to the CISS-21 and ECR-RS, the survey included questions about demographics and lifestyle habits. However, these will not be analyzed in this article.

### Statistical analysis

First, internal consistency of the four parental attachment scales was evaluated by calculating Cronbach’s α and McDonald’s ω coefficients, as adequate reliability of the input variables is a crucial prerequisite for obtaining meaningful and stable cluster solutions (Vargha & Bergman, 2019; Gergely & Vargha, 2021).

To address the issue of high skewness in the attachment scales, the test items were trimmed (with a maximum of 4 set for avoidance items and a maximum of 3 set for anxiety items). With these different value ranges, the scales were then submitted to a POMS transformation (Moeller, 2025).

Next, *k*-means cluster analyses (*k*CAs) were conducted to identify a statistically sound and theoretically interpretable cluster solution of parental attachment. To assess the relationship between coping strategies and the optimal cluster solution of parental attachment, two-way ANOVA was conducted for each coping style as dependent variable with grouping factors of gender and cluster. In addition, the main coping style patterns were explored via cluster analyses, and the obtained optimal cluster solution was related to the attachment classification in a cross-tabulation.

All analyses were carried out using the software packages ROPstat (Vargha et al., 2015) and ROP-R (Vargha & Bánsági, 2022; Vargha & Grezsa, 2024), as well as the SPSS statistical software (version 29.0).

## Results

### Method of ECR-RS scale transformation

The variables in *k*CAs included the four scales of AvoidMo, AnxMo, AvoidFa, and AnxFa. The basic descriptive statistics for these scales are summarized in the upper part of Table 1. As shown here, the original forms of the four variables exhibit highly non-normal distributions. This issue is particularly pronounced with the anxiety scales, where the medians are either equal or very close to the lower scale limits. The highly positive skewness values indicate long right tails in the distributions, suggesting the presence of potentially heterogeneous clusters in these regions.

**Table 1:** Basic descriptive statistics of the four attachment scales (N = 1485).

Original scale	Median	Mean	SD	Minimum	Maximum	Skewness	Kurtosis
AvoidMo	1.83	1.98	0.83	1	5	0.96***	0.74***
AnxMo	1.00	1.49	0.81	1	5	1.96***	3.78***
AvoidFa	2.20	2.39	0.96	1	5	0.68***	-0.08
AnxFa	1.33	1.67	0.94	1	5	1.61***	2.11***

**New scale**	**Median**	**Mean**	**SD**	**Minimum**	**Maximum**	**Skewness**	**Kurtosis**

AvoidMo	1.83	1.94	0.75	1	4	0.65***	-0.27*
AnxMo	1.00	1.41	0.61	1	3	1.36***	0.67***
AvoidFa	2.17	2.29	0.82	1	4	0.31***	-0.79***
AnxFa	1.33	1.53	0.67	1	3	1.00***	-0.34**

*Notation*.

* : *p* < .05

** : *p* < .01

*** : *p* < .001

The basic descriptive statistics for the transformed four scales are summarized in the lower part of Table 1 (New scale). As can be seen, the levels of skewness and kurtosis become substantially lower due to the trimming transformation of the items. Furthermore, we worked with these new scales.

### Reliability

The reliability level of each attachment scale is excellent (see Table 2). Given the high reliability of the four parental attachment scales, these measures were deemed psychometrically suitable for use in CA to explore parental attachment types.

**Table 2:** Cronbach’s α and McDonald’s ω reliability measures of the four attachment scales.

Scale	Cronbach’s α	CI._95_	McDonald’s ω	CI._95_
AvoidMo	0.845	0.833-0.858	0.845	0.831-0.859
AnxMo	0.820	0.804-0.836	0.821	0.799-0.844
AvoidFa	0.844	0.832-0.857	0.844	0.831-0.857
AnxFa	0.826	0.810-0.841	0.827	0.808-0.847

### Clusters of parental attachment

Vargha and Grezsa (2024) performed a series of CA using the same attachment variables on the male subsample of the present study. As these variables had different range scales (see the lower part of Table 1), Vargha and Grezsa *z*-standardized the variables prior to CA. However, in a recent paper, Moeller (2025) drew attention to the pitfalls of *z*-standardization in CA. If the variables have different scales, when some transformation is necessary, to create a common scale Moeller (2025) suggests choosing a better transformation instead of *z*-transformation, such as POMS. After this, variables have always the same range, the [0; 1] interval (Cohen et al., 1999; Gower, 1971).

Hence, we performed POMS transformations on our new parental attachment scales having different ranges, which yielded variables AvoidMoP, AnxMoP, AvoidFaP, and AnxFaP, and conducted *k*-means CAs with them. Vargha and Grezsa (2024) and Vargha (2025) found that an optimal cluster structure was achieved with seven clusters, therefore we conducted *k*-means CAs for k values within the range of 5 to 8 in the present study.

The computed cluster quality coefficient (QC) values of these solutions, summarized in Table 3, show that the overall homogeneity is acceptable for all *k* values (EESS% > 70), but only for *k* > 5 drops the maximal HCmeanS value below 1 (Vargha et al., 2016). For the XBmod and Silhouette coefficient QCs, which measure cluster separability, values above 0.50 are desirable, which is also the case for *k* > 5, although only approximately for XBmod (Bergman et al., 2017). As the QCs do not improve substantially beyond *k* = 6, the 6-cluster *k*-means solution was selected for further interpretation and analysis.

**Table 3:** Some QCs for the k-means solutions for cluster numbers between k = 5 and k = 8.

Cluster number	EESS%	PB	XBmod	Silhouette coefficient	HCmeanS	HCstan min-max
5	70.5	0.44	0.22	0.64	0.591	0.22-1.23
6	75.0	0.44	0.44	0.66	0.503	0.18-0.85
7	77.2	0.42	0.41	0.65	0.459	0.15-0.90
8	78.4	0.40	0.43	0.64	0.434	0.14-0.81

*Note*. EESS% = Explained error sum of square percentage; PB = Cluster point biserial correlation; XBmod = Modified Xie-Beni index; HCmeanS = average standardized within cluster difference.

**Figure 1:**
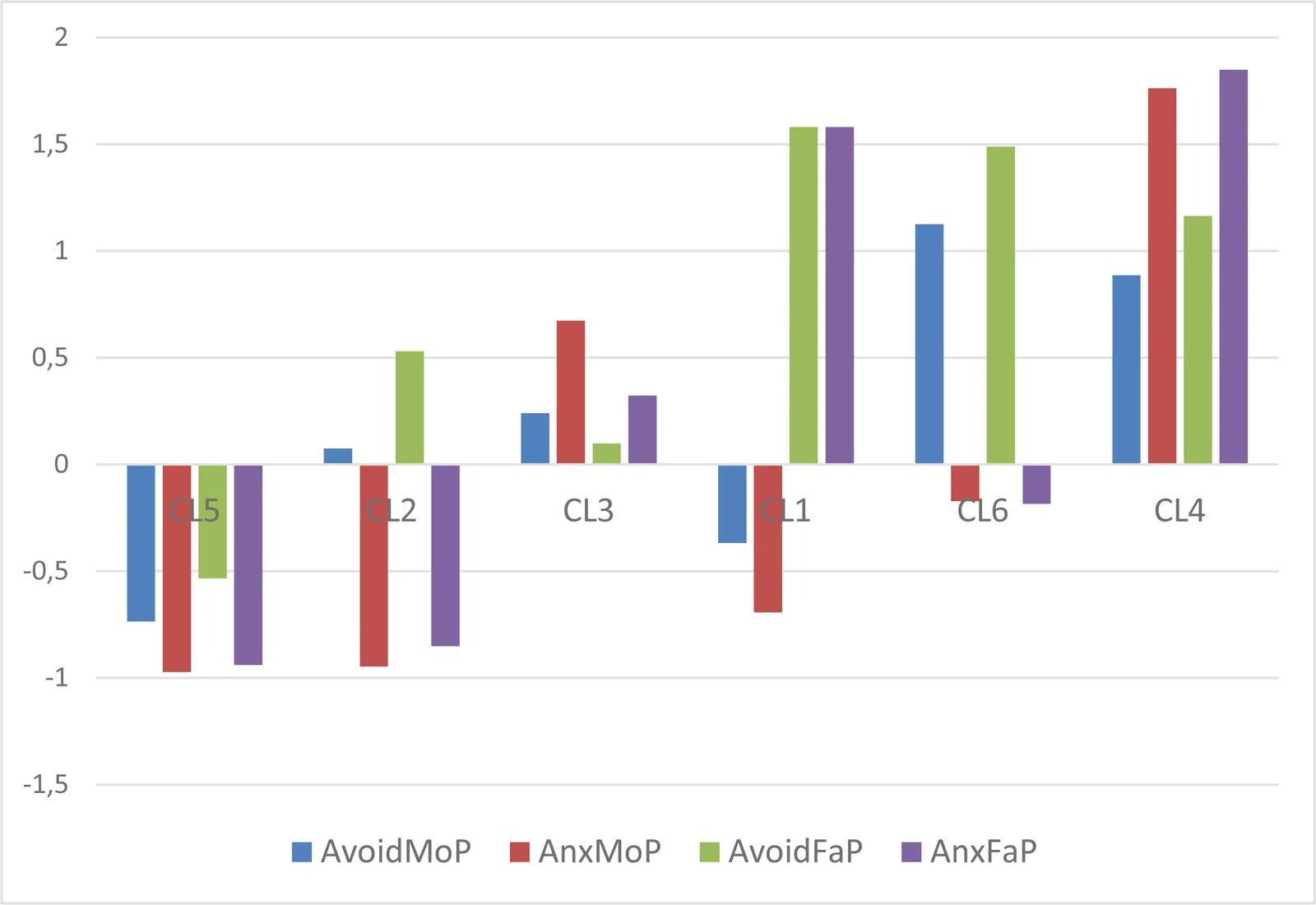
The z_t_-standardized centroid pattern of the 6-cluster k-means solution (clusters arranged in descending order of overall attachment level)

Vargha (2025) suggested using the *z_t_*-standardized cluster means to explain a cluster solution after CA. With normal *z*-standardization we standardize variables separately and differently before CA. With such a transformation, the original meaning of the scale unit is lost, and when calculating distances, the different variables will be weighed in proportion to the inverse of their variance. However, in *z_t_*-standardizing the cluster means we use the total mean *M_t_* of the variables and the *SD_t_*, the square root of the pooled common variance after a CA, and this seems to be useful for explaining centroid patterns (Vargha & Postáné Török, 2026). The pattern of centroids of the optimal 6-cluster *k*-means solution is summarized in Table 4, and the *z_t_*-standardized centroid pattern of the 6-cluster *k*-means solution can be seen in Figure 1.

**Table 4:** The pattern of z_t_-standardized means in the 6-cluster k-means solution (H = High, L = Low; more pluses indicate more extreme means).

Cluster	AvoidMoP	AnxMoP	AvoidFaP	AnxFaP	CLSize	HCstan
CL1	.	L	H++	H++	131	0.83
CL2	.	L	(H)	L	383	0.34
CL3	.	(H)	.	.	166	0.80
CL4	H	H+++	H+	H+++	178	0.85
CL5	L	L	(L)	L	453	0.18
CL6	H+	.	H++	.	174	0.82

Based on Figure 1 and Table 4 we see clearly differentiated attachment patterns with varying degrees of internal homogeneity. The most secure attachment profile is represented by CL5, which is characterized by consistently low levels of avoidance and anxiety toward both parents. In contrast, the most insecure attachment pattern is observed in CL4, where both maternal and paternal avoidance and anxiety are markedly elevated, indicating a globally insecure attachment configuration.

Importantly, most clusters exhibit coherent and interpretable patterns across the four attachment dimensions, with no apparent extreme heterogeneity. The two most homogeneous clusters (with HCstan < 0.35) are CL5 and CL2, are also the two largest (including 56% of the total sample) and demonstrate the most favorable paternal attachment patterns. In CL2, anxiety levels in both the mother and father domains are low, whereas the level of avoidance is close to average. CL3 represents an average in all respects. Beyond these standard types, there are also two clusters of non-trivial attachment types. CL1 shows good maternal attachment and poor paternal attachment, whereas CL6 shows high avoidance coupled with average anxiety in both parental domains.

### Gender impact

To assess the relationship between gender and cluster belongingness a two-way frequency table was created (see Table 5). This relationship was significant (χ^*2*^ = 34.86; *df* = 5; *p* < .001) and a medium-level relationship is indicated by Cramér’s *V* being 0.153 (Cohen, 1988). The gender effect is due to three clusters. Male students dominate in the CL2 cluster, characterized by good parental attachment, and in CL6, characterized by high levels of parental avoidance, while female students dominate in CL1, characterized by strongly negative paternal attachment (see Figure 1 and Table 5).

**Table 5:** The percentage of male and female students in the 6-cluster k-means solution.

Clusters	Male	Female	TOTAL
CL1	38.5	**61.5**	100
CL2	**62.1**	37.9	100
CL3	56.0	44.0	100
CL4	47.5	52.5	100
CL5	49.0	51.0	100
CL6	**61.8**	38.2	100
TOTAL	53.6	46.4	100

### Clusters and coping styles

**Figure 2:**
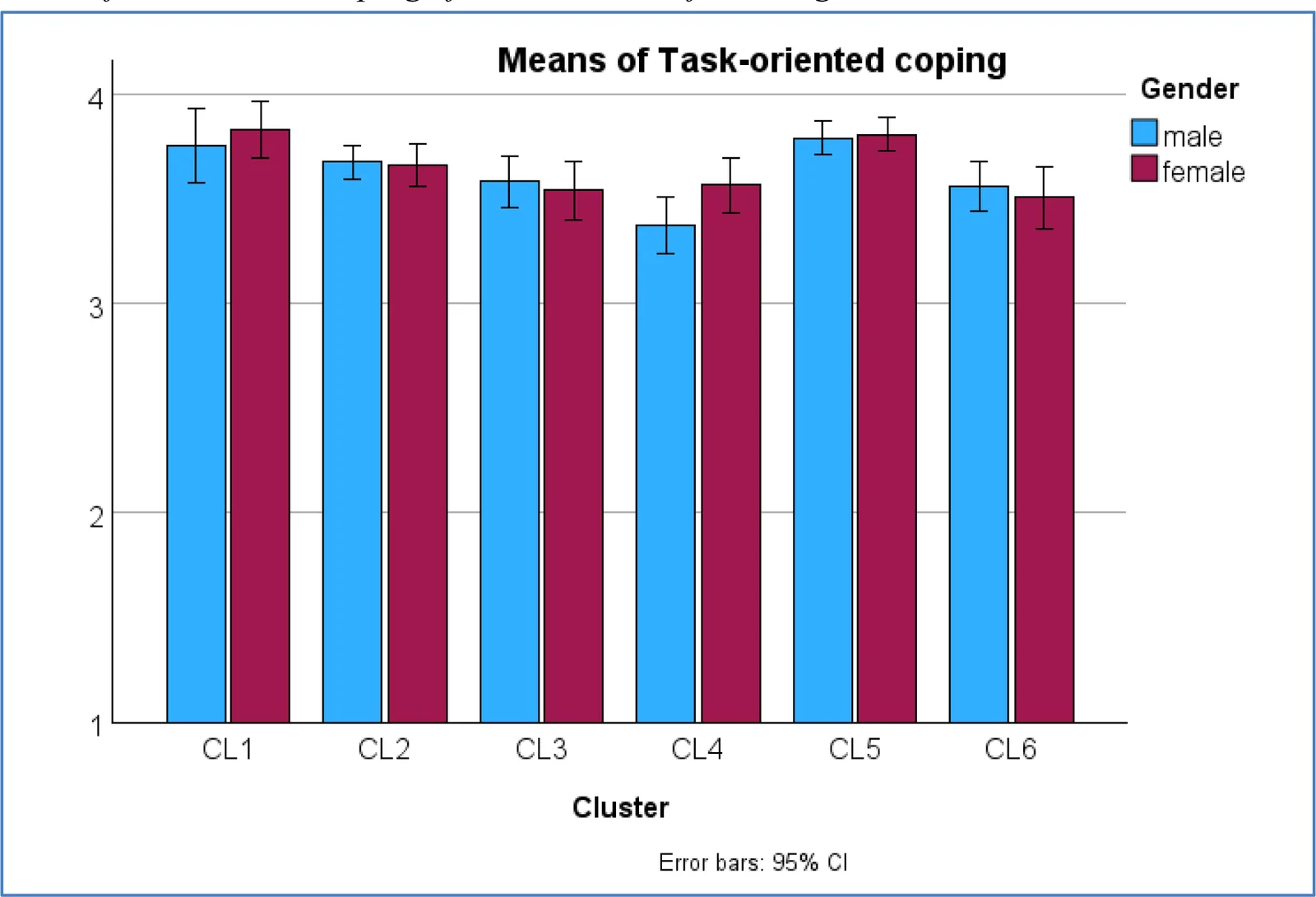
Means of task-oriented coping of the six clusters for both genders

To assess the frequency of the different coping strategies across clusters, two-way ANOVA was conducted for each coping style. With TaskOri as dependent variable the twoway ANOVA revealed a significant main effect of attachment cluster on task-oriented coping (*F*[5, 1388] = 11.16, *p* < .001, *η^2^_p_* = .039), indicating that adolescents’ use of taskoriented stress management strategies differed across parental attachment types (see Figure 2). Neither the main effect of gender (*p* = .440) nor the gender by cluster interaction (*p* = .426) was significant. Post hoc analyses – conducted using Scheffé’s contrast method – showed that one cluster (CL5, representing the most secure attachment type) exhibited a significant above average level (*F*(5; 1388) = 6.01, *p* < .001), and one cluster (CL4, representing the most insecure attachment type) was significantly below average (*F*(5; 1388) = 3.17, *p* = .008; see Figure 2).

**Figure 3:**
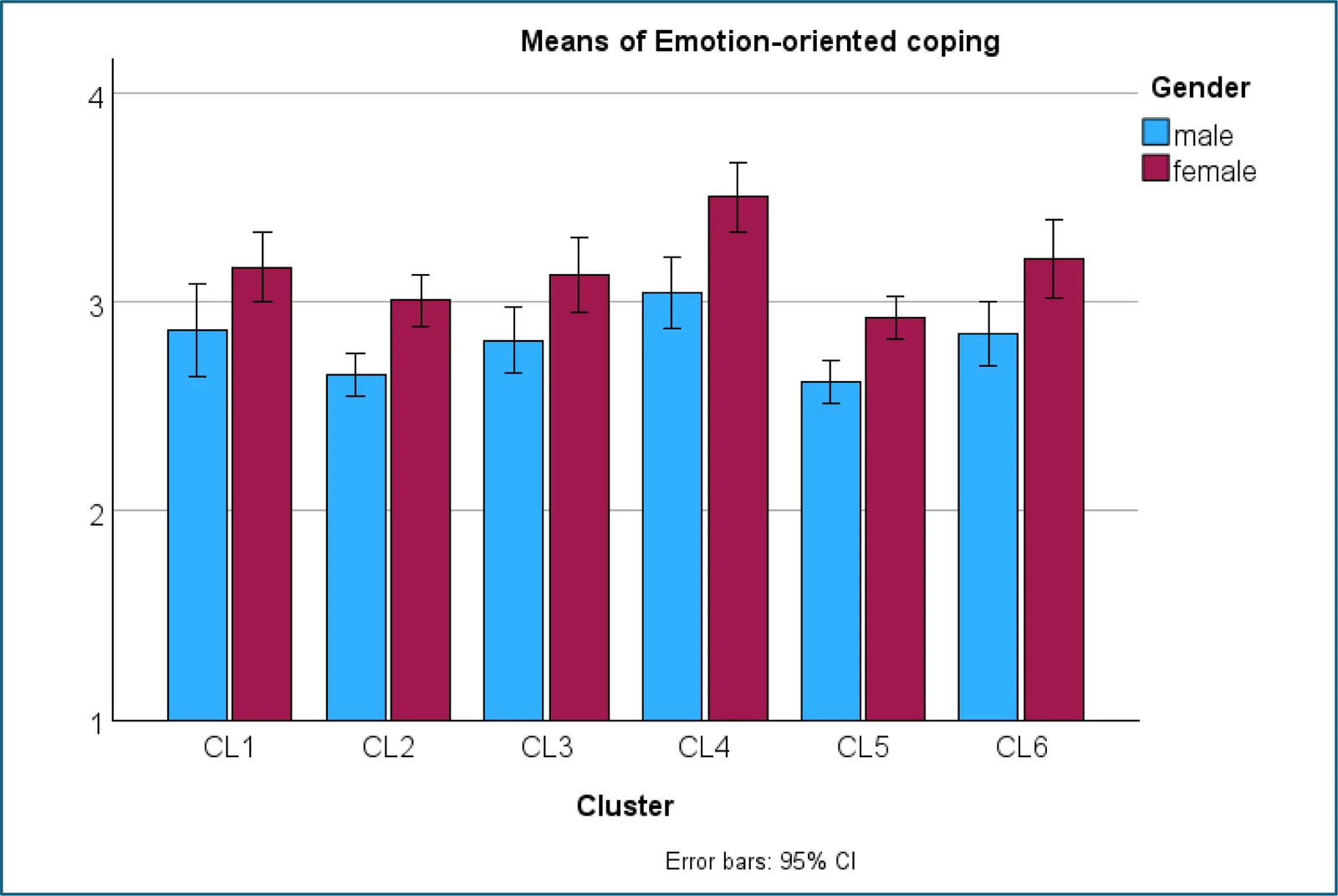
Means of emotion-oriented coping of the six clusters for both genders

With EmotOri as dependent variable two-way ANOVA revealed significant main effects of gender with a higher level of female students (*F*[1, 1382] = 57.19, *p* < .001, *η^2^_p_* = .040; see Figure 3), and cluster (*F*[5, 1382] = 12.01, *p* < .001, *η^2^_p_* = .042) on emotion-oriented coping. The gender by cluster interaction was not significant (*p* = .939). Post hoc analyses showed that one cluster (CL4, representing the most insecure attachment type) exhibited a significant above average level (*F*(5; 1382) = 5.71, *p* < .001), and two clusters (CL5 and CL2, both representing a good attachment type) was significantly below average (Cl5: *F*(5; 1382) = 6.12, *p* < .001; CL2: *F*(5; 1382) = 2.95, *p* = .012; see Figure 3).

**Figure 4:**
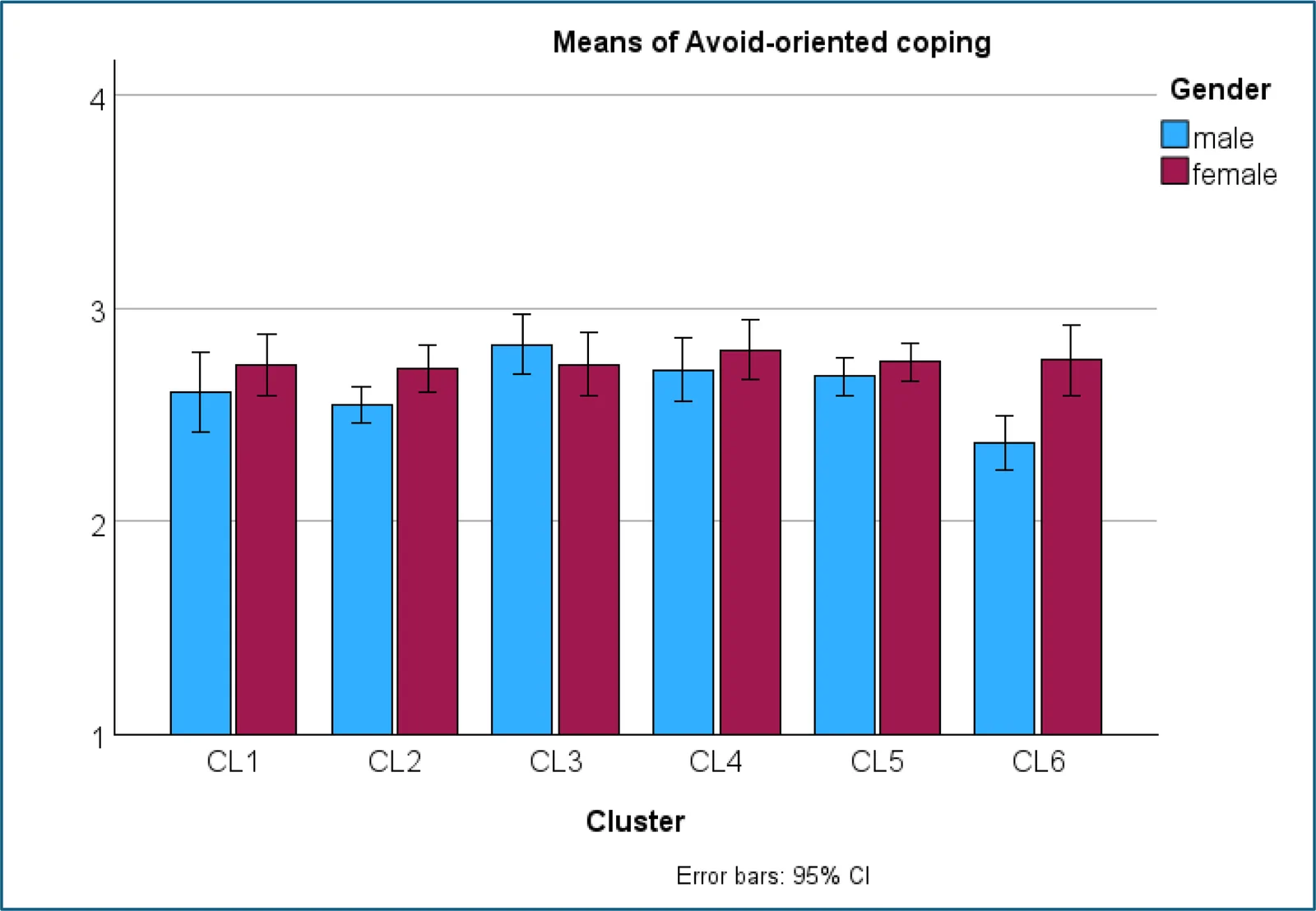
Means of avoidance-oriented coping of the six clusters for both genders

With AvoidOri as dependent variable the two-way ANOVA revealed significant main effects of gender with female dominance (*F*[1, 1376] = 9.85, *p* = .002, *η^2^_p_* = .007; see Figure 4), and cluster (*F*[5, 1376] = 2.85, *p* = .015, *η^2^_p_* = .010) on avoidance-oriented coping. The gender by cluster interaction was also significant (*F*[5, 1376] = 2.32, *p* = .041, *η^2^_p_* = .008), possibly because in one cluster (CL3) male students exhibited higher levels of avoidance-oriented coping than female students, whereas in the other clusters (especially CL6) female students exhibited higher levels of avoidance-oriented coping than male students (see Figure 4). Post hoc analyses conducted using Scheffé’s contrast method did not produce any significant results.

In addition to ANOVAs, we investigated the relationship between coping under stress and the obtained attachment profiles by exploring the main coping style patterns via cluster analysis, and cross-tabulating the obtained optimal cluster solution with the explored 6-cluster attachment classification. Using TaskOri, EmotOri, and AvoidOri as input variables, first we ran Ward-type hierarchical CA and compared a number of quality coefficients (EESS%, XBmod, HCmean, HCmax) of the different solutions for number of clusters between *k* = 3 to 12. Because the means of the three input variables differed strongly from each other, we standardized the variables before performing CA. Based on the hierarchical CA results, we chose *k* = 8 as an optimal number of clusters. Next, we ran a *k*-means CA for *k* = 8 and obtained acceptable QCs (EESS% = 66.3, XBmod = 0.48, Silhouette coeff. = 0.55, HCmean = 0.68, HCmax = 1.11). Without going into technical details, the following conclusions could be drawn from the obtained results.

1. The “High in TaskOri, low in EmotOri, and low in AvoidOri” coping profile, which can be considered a good coping style, occured less frequently than expected in CL4 (the most insecure parental attachment type) and more frequently than expected in CL5 (the most secure parental attachment type).
2. CL4 could also be characterized by a rare use of the “Average in TaskOri, slightly high in EmotOri, and low in AvoidOri” coping profile, being also a good coping style.
3. CL2, representing a good parental attachment type with a low level of parental anxiety could be characterized by the frequent use of the “Average in TaskOri, slightly high in EmotOri, and low in AvoidOri”, and a rare use of the “High in TaskOri, high in EmotOri, and average in AvoidOri” coping styles.

## Discussion

The present study examined the relationship between parental attachment (measured by ECR-RS) and stress management strategies (measured by CISS-21) in adolescence in a person-oriented approach. With the four parental attachment variables (mother and father attachment and anxiety scales) a 6-cluster structure could be identified as the best solution. The main types of parental attachment explored (see Figure 1) were coherent and psychologically meaningful. One homogeneous cluster (CL5; 30.5% of the sample) represented the most secure type of parental attachment with low levels of avoidance and anxiety, and one (CL4; 12%) represented the most insecure with elevated levels in all variables. In another homogeneous cluster of good parental attachment (CL2; 25.8%), anxiety levels in both the mother and father domains were low, whereas the level of avoidance was close to average. One cluster (CL3; 11.2%) was average in all respects, with a slight increase of parental anxiety. Beyond these standard types, there were also two clusters of non-trivial attachment types. One of them (CL1; 8.8%) showed good maternal attachment and poor paternal attachment, and the other (CL6; 11.7%) showed high avoidance coupled with average anxiety in both parental domains (see Figure 1).

When we examined the relationship between gender and cluster profiles, we discovered that female students predominantly occupied one cluster (CL1). This cluster was characterized by poor paternal attachment alongside positive maternal attachment. However, male students were overrepresented in clusters CL2 and CL6, where the scales of parental avoidance were substantially higher than the scales of parental anxiety. This type of male dominance was also obtained in Vargha and Postáné Török (2026) in an independent sample (young adults aged between 20 and 35). In this study, the same four parental attachment scales were used, but no transformation was performed on them. Despite this difference, there is a high similarity between the explored cluster structures of the two studies. The patterns of four clusters (CL5, CL2, CL1 and CL4 in our study) are almost or completely identical. One cluster (CL6 in our study) shows partial agreement, while only one pair of clusters (CL3 in our study) do not match. This strong correspondence confirms the stability of the explored attachment types. However, it remains to be seen which form of variable (transformed or not, standardized or not) is most appropriate for analysis in CA.

Coping modes were differentially sensitive to attachment configuration across all clusters. In line with the literature (Manolov & Stoyanov, 2025), high-level attachment (CL5) was associated with a high level of task-oriented coping, while low-level attachment (CL4) was associated with a low level of task-oriented coping, regardless of gender. In line with these results, high-level attachment (CL5 and CL2) could be characterized by favorable coping styles and lowlevel attachment (CL4) by unfavorable coping styles.

Like previous findings, emotion-oriented coping displayed a robust and consistent female-greater-than-male effect across clusters (Frydenberg & Lewis, 1993; Cosway et al., 2000; Rafnsson et al., 2006; Eschenbeck, Kohlmann, and Lohaus 2007; Matud, 2004). Avoidance-oriented coping was also dominant among female students, occupying an intermediate position, with modest gender differences. These results indicate that gender effects are coping-specific rather than global, and that attachment insecurity primarily alters the balance among coping strategies (Kobak & Sceery, 1988; Allen et al., 2018).

Attachment security was associated with the most adaptive coping configurations. The globally secure cluster (CL5) exhibited the lowest emotion-oriented coping, the highest task-oriented coping, and average avoidance-oriented coping, suggesting efficient emotional regulation combined with active and effective problem solving. This finding is consistent with the results of Mikulincer and Shaver (2007), who found that adolescents with a sense of security can approach stressful situations with confidence and engage in adaptive coping strategies. This is consistent with attachment theory, which suggests that greater attachment insecurity is associated with an increased reliance on emotionally focused stress responses.

In contrast, the globally insecure cluster (CL4) showed the most maladaptive profile, characterized by markedly elevated emotion-oriented coping, reduced task-oriented coping, and average avoidance. This constellation reflects heightened emotional reactivity and reduced goal-directed stress management. Our findings are accordant with theoretical models that propose adolescents with insecure attachment experience greater challenges in emotional regulation. Consequently, they are more likely to adopt avoidance-oriented or emotion-focused coping strategies, which could exacerbate stress and negatively impact psychological wellbeing (Kobak & Sceery, 1988).

Importantly, the present findings indicate that gender and attachment configuration do not function as independent predictors of coping. Instead, they form functionally integrated regulatory patterns in which attachment-related expectations influence the expression of gender-typical coping tendencies under stress. From an attachment perspective, internal working models provide adolescents with implicit expectations about the availability of support, the effectiveness of emotional expression, and the controllability of stressful situations (Bowlby, 1988; Mikulincer & Shaver, 2007). These expectations appear to organize how gender-linked coping tendencies are mobilized and combined.

Although females consistently reported higher levels of emotion-oriented coping, we cannot declare that this is uniformly maladaptive. In secure or low-anxiety attachment configurations, emotional coping co-occurred with high task-oriented coping, suggesting that emotional engagement may serve a regulatory or preparatory function, facilitating effective problem solving (Lazarus & Folkman, 1984; Compas et al., 2001). In contrast, in insecure attachment configurations, elevated emotional coping was less effectively integrated with task-oriented strategies, increasing the likelihood of internally focused distress rather than adaptive regulation (Kobak & Sceery, 1988; Mikulincer & Shaver, 2007).

Similarly, the result suggest that attachment security may override gender-related variability in problem-focused coping, enabling both male and female adolescents to mobilize active strategies when stressors are perceived as manageable. Together, these findings support a contextualized view of gender differences in coping, emphasizing that coping strategies derive their functional meaning from the attachmentbased relational context in which they are enacted.

These patterns may be particularly salient in adolescence, a developmental period characterized by heightened emotional reactivity, increasing autonomy demands, and stillmaturing self-regulatory capacities (Compas et al., 2001).

During this phase, attachment-related internal working models may play a central role in organizing coping behavior, compensating for limited regulatory resources (Kobak & Sceery, 1988; Allen et al., 2018). Consequently, attachmentbased coping styles may function as transitional regulatory strategies, shaping the consolidation of adult coping and emotion-regulation styles over time (Compas et al., 2001; Mikulincer & Shaver, 2019).

From an applied perspective, the findings suggest that interventions aimed at improving adolescent coping may be more effective when they are tailored to attachment-based profiles rather than targeting isolated coping dimensions. Because coping strategies operate within broader attachment-related regulatory systems, interventions focusing solely on increasing or decreasing specific coping behaviors (e.g., reducing emotional coping or promoting problem solving), may overlook the relational expectations how these strategies are deployed under stress.

In summary, the results demonstrate that parental attachment patterns are associated with qualitatively distinct coping configurations in adolescence. These findings highlight the importance of attachment-informed intervention approaches that strengthen existing adaptive capacities while addressing specific regulatory limitations. Such tailored strategies may enhance adolescents’ ability to flexibly deploy coping responses across contexts, thereby supporting more stable and adaptive stress regulation during a critical developmental period.

## Data Availability

The data that support the findings of this study are available upon reasonable request from the corresponding author.

## Action editor

Lars-Gunnar Lundh served as action editor for this article.

## Open access

This article is distributed under the terms of the Creative Commons Attribution 4.0 International License (http://creativecommons.org/licenses/by/4.0/), which permits unrestricted use, distribution, and reproduction in any medium, provided you give appropriate credit to the original author(s) and the source, provide a link to the Creative Commons license, and indicate if changes were made.
